# Health and care service utilisation and cost over the life-span: a descriptive analysis of population data

**DOI:** 10.1186/s12913-020-05295-2

**Published:** 2020-05-19

**Authors:** Jorid Kalseth, Thomas Halvorsen

**Affiliations:** Department of Health Research, SINTEF Digital, P.O. Box 4760, Sluppen, NO-7465 Trondheim, Norway

**Keywords:** Health care costs, Health services, Long-term care, Hospital, Nursing home, Home care, Utilisation, Age pattern, Gender differences

## Abstract

**Background:**

Current demographic changes affect both the level and composition of health and care needs in the population. The aim of this study was to estimate utilisation and cost for a comprehensive range of health and care services by age and gender to provide an in-depth picture of the life-span pattern of service needs and related costs.

**Methods:**

Data on service use in 2010 for the entire population in Norway were collected from four high-quality national registers. Cost for different services were calculated combining data on service utilisation from the registries and estimates of unit cost. Data on cost and users were aggregated within four healthcare services and seven long-term care services subtypes. Per capita cost by age and gender was decomposed into user rates and cost per user for each of the eleven services.

**Results:**

Half of the population is under 40 years of age, but only a quarter of the health and care cost is used on this age group. The age-group of 65 or older, on the other hand, represent only 15% of the population, but is responsible for almost half of the total cost. Healthcare cost dominates in ages under 80 and mental health services dominates in adolescents and young adults. Use of other healthcare services are high in middle aged and elderly but decreases for the oldest old. Use of care services and in particular institutional care increases in old age. Healthcare cost per user follows roughly the same age pattern as user rates, whereas user cost for care services typically are either relatively stable or decrease with age among adults. Gender differences in the age pattern of health and care costs are also revealed and discussed.

**Conclusion:**

The type of services used, and the related cost, show a clear life-span as well as gender pattern. Hence, population aging and narrowing gender-gap in longivety calls for high policy awarness on changing health and care needs. Our study also underscores the need for an attentive and pro-active stance towards the high service prevalence and high cost of mental health care in our upcoming generations.

## Background

Demographic changes affect both the level and composition of health and care needs in the population. The aging populations of the western world have spurred interest in the age distribution and age profiles of healthcare cost [1]. The health registries of the Nordic countries are known for their quality and high coverage. This study is the first to take advantage of population registries in Norway to provide estimates for a comprehensive set of these distributions. The ability to include population data on long-term care services for most publicly funded services is a major strength of this study. Including long-term care services is particularly important since age effects are likely to be most pronounced for these services [2]. Furthermore, Norway, like other Nordic countries, has an universal, extensive and mainly publicly funded health and care service system [3], which means that there are extensive service offerings for all in need of health related care, regardless of age. This makes this study uniquely positioned to make reliable estimates of the age profiles and age distribution of health and care cost.

Our aims with this study are threefold. First, to identify the age-groups with the highest health and care cost. Second, to describe the change in service composition as age increases; and, third, to describe user rates (users per capita) and average cost per user, addressing the question whether age differences in average cost relate to variation in user rates, cost per user or both.

Analyses are done separately for males and females since there are systematic gender differences in health and care services consumption.

## Methods

### The setting

Responsibility for health and long-term care services are split between the state and the local government in Norway. Specialist (secondary) healthcare is the responsibility of four state owned regional health authorities (RHAs). Most secondary healthcare services are run by public health enterprises owned by the RHAs and some by private providers under contract with the RHAs. Primary healthcare and long-term care is the responsibility of Norway’s 422 municipalities (2019). Primary care physicians are commonly organized in private group practices, working under contract with their local municipality, with a gatekeeping function for referral to specialist healthcare. Municipal nursing and care services (long-term care) are mostly provided by public (municipal) providers. Most health and long-term care expenditures are publicly financed, but some co-payment for outpatient primary and specialist health services, as well as home care (practical aid etc.) and long-term residential care, do exist. All in all, user charges cover about 15% of total health expenditures [4] and 7% of expenditures in municipal nursing and care services [5].

### Data sources

Data on service utilisation were collected from four public registries. Data for primary healthcare physicians, comprising general practice and emergency visits, and data for self-employed specialists working under contract with an RHA were collected from the registry for Control and Payment of Health Refunds (KUHR). It covers healthcare activities that are eligible for partly reimbursement from the National Insurance Scheme. Inpatient and outpatient specialist (secondary) healthcare data including somatic services, psychiatric services and substance abuse treatment were collected from the Norwegian Patient Registry (NPR). Data on long-term care services were obtained from the Norwegian Information System for the Nursing and Care Sector (IPLOS database). Finally, data on prescription medicine were retrieved from the Norwegian Prescription Database (NorPD), covering all dispensed prescription medicine from farmacies. Medicine cost in hospitals and nursing homes are included in the cost estimates for the respective services. Data for the entire population in 2010 was retrieved from these four registries. In most cases the service registries include information on utilisation and not cost. Cost was calculated by multiplying the chosen unit of utilisation with an estimate of unit cost. The details on cost calculations, as well as services not covered in this study, are given in the Supplemental material.

The following service categories, classified either as healthcare or long-term care, were used in the analyses:

*Healthcare* (HC): primary care (physicians), prescription medicine, specialist somatic healthcare, and mental healthcare (comprising specialist psychiatric and substance abuse treatment).

*Long-term care* (LTC): long-term residential (nursing home) care, short-term stays in nursing homes (including short stays for examination/treatment, (re) habilitation, other short stays, day-time and night-time stays), home nursing, home help (including practical aid, and training of daily activities, user-controlled personal assistance, meals on wheels and safety alarm), care salary, respite care, activity services (including support persons and day activities).

The number of unique users (persons) in 2010 were identified for each of the 11 service categories based on unique personal identifier contained in the registries.

Age was calculated based on year of birth. Five-year age-bands were used (0–4, 5–9, 10–14 etc), with the exception of centenarians (100 years or more) comprising one group.^1^ In calculating per capita cost and user rates, the denominator for the total population is based on population data by age group collected from Statistics Norway.

### Analyses

The study provides descriptive analyses (tables and graphics) of HC and LTC utilisation and cost by age and gender which results in five types of estimates:

1. *Costs and number of users by service type*
2. *The distribution of cost and service users by age group*
3. *The within age-group composition of cost by service type*
4. *The age profile of average (*per capita*) total cost, HC cost, LTC cost, and by service type*
5. *The age profiles of user rates and cost per user by service type*

Data for groups with less than 30 users (for either one or both genders) are not shown.

## Results

### Total cost by service, gender and age-group

The total estimated cost of the services included in the study were about 172 billion NOK in 2010 (see Table 1), amounting to 34,500 NOK per capita. Assuming that these expenditures grow with the same rate (in current prices) as total healthcare (as measures by System of Health Accounts (SHA)), the 2018 figures would be 268 billion NOK or about 53,800 NOK per capita.

**Table 1:** Population, estimated cost and number of users by service category, age and gender

				Distribution (%) by						
				Gender		Age group				
				F	M	0–19	20–39	40–64	65–84	85+
**Population**	4.993 million			50.0	50.0	25.1	26.6	32.8	13.0	2.6
					F^a^	48.7	49.0	48.9	53.5	68.3
					M^a^	51.3	51.0	51.1	46.5	31.7
**Costs**	Billion NOK	% of Total	% of HC/LTC	F	M	0–19	20–39	40–64	65–84	85+
Total (THC)	172.3	100.0		55.8	44.2	8.7	17.6	27.9	27.6	18.3
					F^b^	44.6	53.3	50.0	54.7	74.0
					M^b^	55.4	46.7	50.0	45.3	26.0
Healthcare (HC)	102.2	59.3	100.0	52.6	47.4	11.3	20.8	34.3	27.4	6.3
- Somatic	59.6	34.6	58.3	52.6	47.4	9.6	14.0	32.4	35.2	8.7
- Mental health	21.9	12.7	21.5	51.1	48.9	18.2	41.0	33.4	6.5	0.9
- GP	7.5	4.3	7.3	57.5	42.5	12.7	24.0	36.3	21.8	5.2
- Medicine	13.2	7.7	12.9	52.1	47.9	6.7	15.8	42.9	29.7	4.9
Long-term care (LTC)	70.1	40.7	100.0	60.5	39.5	4.9	12.9	18.5	27.9	35.8
- Activity	2.4	1.4	3.4	54.7	45.3	10.4	25.6	32.0	18.6	13.4
- Home help	17.1	9.9	24.4	49.2	50.8	3.4	34.0	40.7	13.1	8.9
- Care salary	0.8	0.5	1.2	42,0	58.0	45.4	19.8	17.7	13.9	3.3
- Respite care	2.5	1.5	3.6	37.3	62.7	73.3	10.8	3.7	8.1	4.1
- Home nursing	16.9	9.8	24.1	60.3	39.7	1.8	11.9	22.5	31.8	32.0
- Short stay	4.6	2.7	6.6	61.5	38.5	0.2	0.6	7.1	45.9	46.3
- Long stay	25.7	14.9	36.7	71.2	28.8	0.3	0.4	3.5	35.2	60.6
**Users**	Number (1000)	% of Population		F	M	0–19	20–39	40–64	65–84	85+
Healthcare (HC)										
Somatic	1936	38.8		55.4	44.6	18.3	23.2	33.9	20.4	4.2
Mental health	226	4.5		53.8	46.2	26.1	34.6	32.9	5.4	1.0
GP	3887	77.8		53.4	46.6	22.2	25.7	33.9	15.3	3.0
Medicine	3353	67.2		55.0	45.0	17.3	25.4	36.6	17.5	3.1
Long-term care (LTC)	314	6.3		62.6	37.4	5.7	9.9	17.5	35.7	31.2
Activity	54	1.1		56.4	43.6	16.2	18.3	26.0	22.7	16.8
Home help	173	3.5		68.0	32.0	0.9	6.9	15.2	39.6	37.4
Care salary	11	0.2		42.0	58.0	44.6	15.8	16.3	17.6	5.6
Respite care	16	0.3		38.9	61.1	56.9	10.2	4.8	17.6	10.5
Home nursing	189	3.8		61.8	38.2	1.5	10.8	20.9	36.9	29.8
Short stay	51	1.0		61.7	38.3	0.3	0.6	7.1	46.5	45.5
Long stay	48	1.0		69.2	30.8	0.3	0.5	3.4	35.0	60.9

^a^Gender distribution of population within age-groups. ^b^ Gender distribution of total cost within age-groups

The relative size of different services depend on whether you look at how many persons use the service or their share of total cost. Primary care and prescription medicine, for instance, are used at least once by most people (> 2/3) during a year, but their shares of total cost were only 4,3% and 7,7% respectively. Long-term care services, on the other hand, were used by about 6% of the population, but amounts to 40% of total cost.

People under 40 constitutes more than half of the population in 2010, but incurred only about ¼ of cost, while people over 85 constitutes less than 3% of the population, but accounted for 18% of cost. The concentration of spending in old age reflects the distribution of spending on LTC. People aged 85+ accounts for more than 35% of LTC cost, while the age group 80+ years are responsible for almost 50% of the cost (not shown in table).

Females have higher share of costs than males, more so for LTC (60,5%) than HC (52,6%), reflecting that females live longer than men; the share of females in the population is almost 70% among the 85 years and older; and that females for most age-groups constitutes a higher share of total cost than of population, the exception being among children and adolecents. For most services the gender distribution of costs and users are relatively similar with a notable exeption for home help where females constitutes less than 50% of costs but almost 70% of users.

**Fig. 1:**
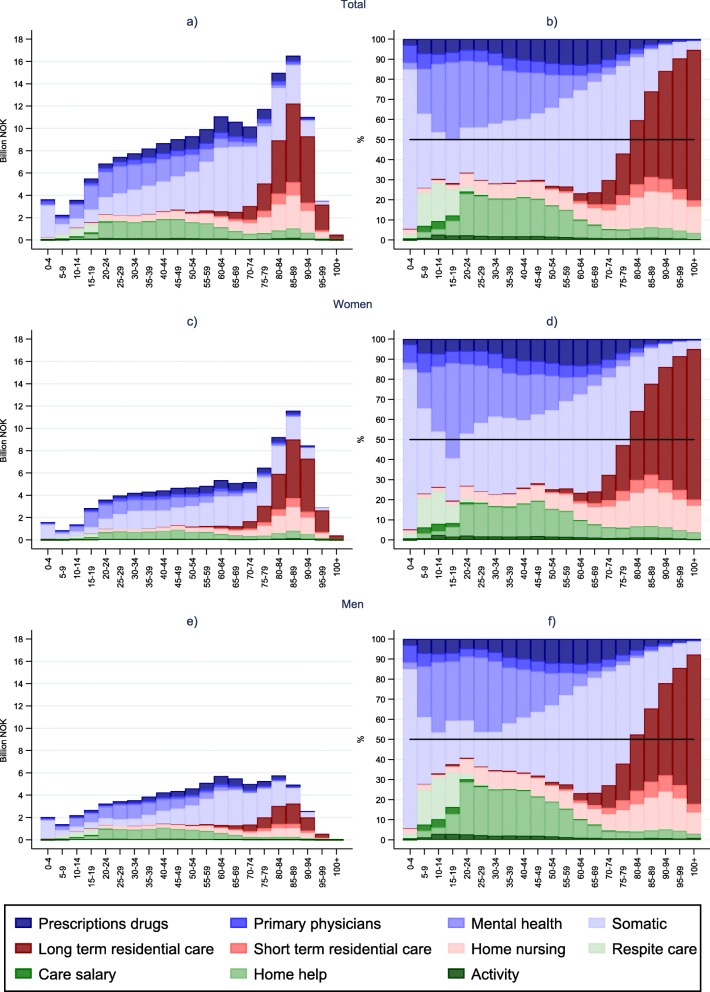
Health and long-term care cost by age. Billion NOK and composition (%).

The panels to the left in Fig. 1 show the absolute spending for 5-year age-bands. Highest spending is on 85–89 year olds, followed by 80–84. This reflects the spending on elderly females, who outnumber males to an increasing degree in high age. Among males, total spending were highest in age-groups 60–64 and 80–84.

The panels to the right in Fig. 1 show how the within age-group composion of cost by type of service vary with age. In the youngest age group (0–4) somatic cost for newborn dominates. In childhood and adolescence the somatic share decreases, and social care services, e.g. related to parental care (i.e. care salary and respite care), increases. Most striking, however, is the importance of mental health services in these ages. It constitutes nearly half of total cost among girls aged 15–19. This pattern continues into young adulthood, and the share of cost for mental health is actually higher than for somatic health in the age groups between 10 and 25 years for both genders. In early adulthood, when most young people leave their childhood home, the type of social LTC services change. For young adults with care needs, home help services become important, and the dominace of social^2^ care in LTC continues well into middel age. HC costs constitute the bigger share of total cost for ages under 80 and the share of costs related to somatic health and prescription medication increases in middle aged. This is particular evident for males. For females, somatic and primary care costs rise in their childbearing years. Approaching old age, an increasing share of LTC cost is tied to home nursing and residental care. LTC nursing cost becomes more dominant in high age, while the HC share of cost decreases. Residential care costs dominate among the oldest old; after 85 years of age half or more of total cost were related to nursing home care, increasing to nearly 80% in the 100+ age group.

### Per capita costs

**Fig. 2:**
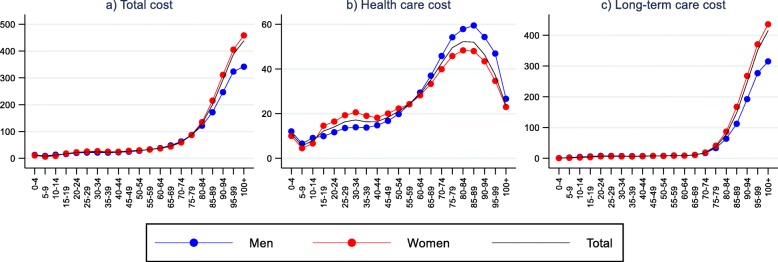
Per capita costs by age and gender. Total cost, healthcare cost, and long-term care cost

Age patterns of per capita cost are shown in Fig. 2 for total, HC and LTC cost. Total per capita cost increases with age, from about NOK 10,000 to almost 40,000 in the age groups under 65, and accelerates after 65, but more so for females than for males. Compared to the age group 60–64, cost in the 100+ age group were 9 times higher among males and and 12 times higher for females. The age pattern of total per capita cost reflects the age pattern of LTC cost, especially among elderly. LTC cost in the 100+ age group were 50 times higher among females and 35 times higher among males compared to the age group 60–64. Per capita HC cost also increases with age until late 80s, but for these costs there are a substantial decrease for the oldest age group. Average HC cost at age 84–89 is approximately 2 times higher than in the 60–64 age band.

**Fig. 3:**
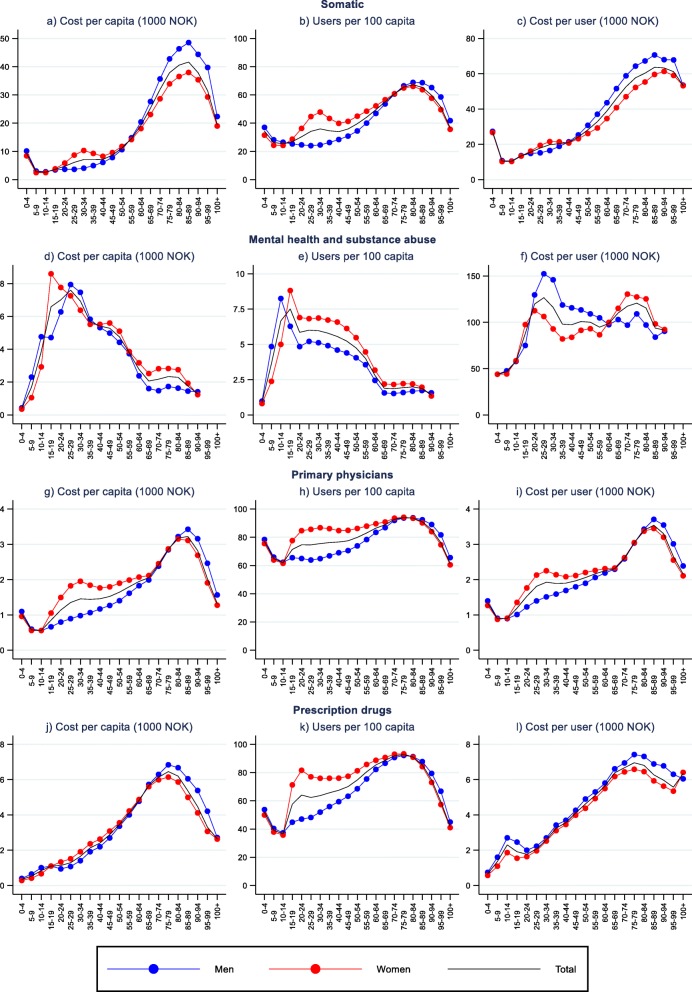
Decomposition of per capita cost of different healthcare services, by age and gender

**Fig. 4:**
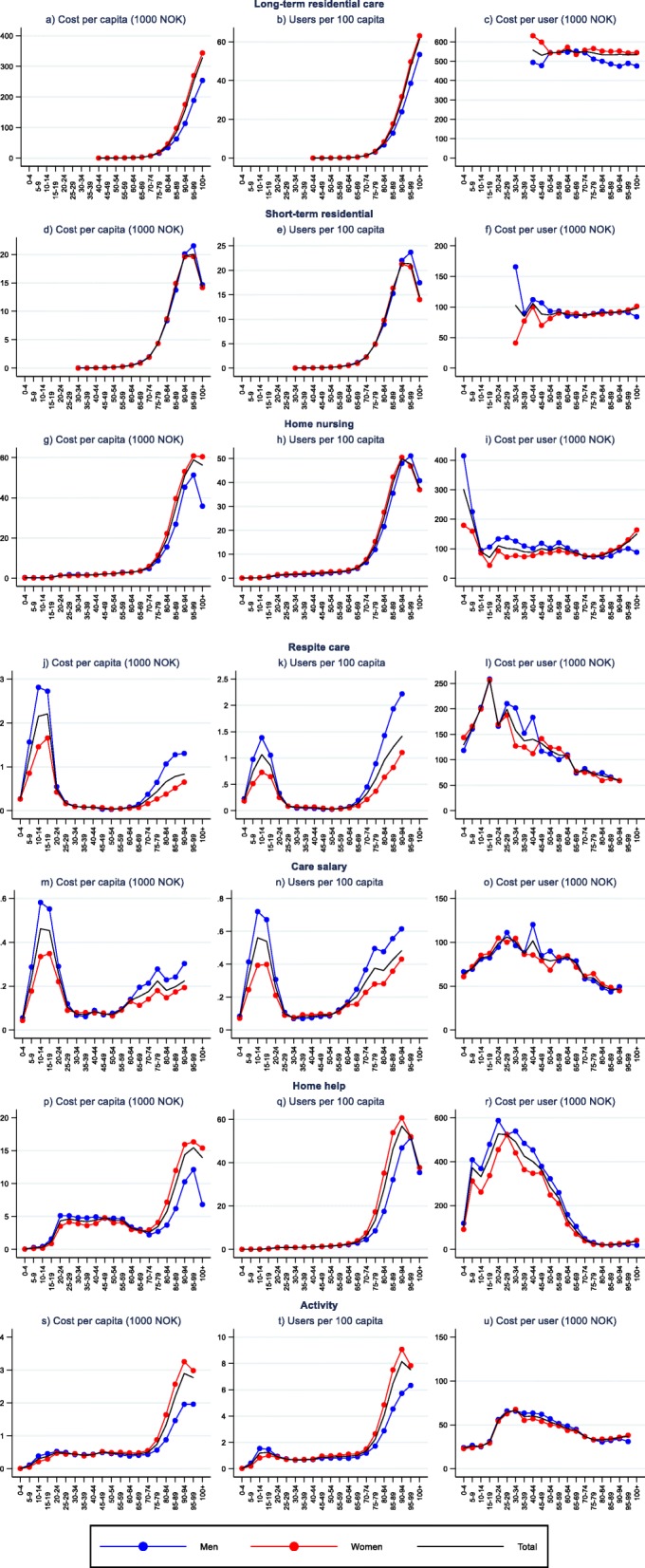
Decomposition of per capita cost of different long-term care services, by age and gender

Figures 3 and 4 display per capita cost for the specific HC and LTC services. The age pattern for somatic health, primary care and prescription medicine are quite similar (Fig. 3). The notable exceptions to the overall pattern for HC services are first and foremost a very different age pattern for mental health, for which per capita cost peakes in the late teens for females (in particular) and late 20s for males. Moreover, females in their 30s and 40s have a non-increasing age profile for primary care and somatic care after the initial steep increase in late teens and twenties. Since we use five-year age-bands, the high per capita HC cost among the new-born is masked.

For most LTC services (Fig. 4), per capita costs are relatively low and stable over many age-bands, but increase substantially into old age. Respite care and care salary deviate from this pattern with highest per capita cost in children and adolescents.

### Decomposition of per capita cost: user rates and cost per user

Figures 3 and 4 display the decomposition of per capita costs of specific services into user rates and cost per user. For somatic health, primary care, and prescription medicine, age patterns of both user rates and user cost resemble the age patterns of per capita cost. Differences in cost per user, however, contribute more to the observed age differences in per capita costs than differences in user rates. For mental health it is the other way around.

For LTC services the age patterns of per capita costs mirrors to a large degree the age patterns of user rates. Cost per user are relatively stable over the age groups for residential services. User costs of home nursing are also stable among adults until their late 50s, from which user costs decrease until they start increasing in higher age (80+). For other LTC services user cost are highest in the teens and young adulthood, and decrease until old age where it stabilises. Long stay residential care and home help for young adults are associated with the highest user costs.

Gender differences in user rates and user costs are evident. User rates are higher for females than males for HC among youths and non-elderly adults, as well as for long-term residential care, activity and, except similar rates for the two oldest age-groups, home-help for elderly. For instance, females have almost double the rate of somatic healthcare compared to males at 30–34 years, and twice the user rate for home help in the late 70s to early 80s. Men, on the other hand, have, with the exception of mental health, higher user rates for HC among elderly, for respite care and care salary, and for home nursing and short residential stays for the two oldest groups (≥95).

Females also have higher user costs for primary care for ages between 15 and 70 and for somatic health services for ages between 20 and 40 years. The same goes for long-term residential care and home nursing in high age. Males, on the other hand, have higher user cost for prescription medicine in most age groups, for home help and home nursing among non-elderly, for somatic health in the age group 50–90 years, for mental health in ages between 15 and 70 and in high age (≥85) for primary care.

Age patterns are relatively similar for males and females for most services. An important exception is home nursing, for which females have higher user costs in old age than in younger ages while the opposite is observed for males. All in all, gender differences in user rates and user costs lead to higher costs for females than males for most services, the female share of cost varying from less than 40% for respite care to over 70% for long-term residential care.

## Discussion

Using high quality individual level data on HC and LTC service utilisation from four different national registries covering the entire Norwegian population we were able to estimate age and gender profiles of total and disaggregated HC and LTC costs as well as utilsation rates and costs per user of different services. The data contained information on more than 3.9 million users of primary care, 3.3 million users of prescription drugs, 1.9 million users of specialist somatic services, 226 thousand users of specialist mental health (psychiatric and substance abuse treatment) services, and 314 thousand users of LTC services. Our costs estimates correspond well to the national costs of the included services as measured in the System of Health Account (SHA) or other sources (see Table S3 in Supplemental materials). Our cost estimate does not include all health services and health costs included in SHA, e.g. physiotherapy, dental and optician services, prevention and public health services and health administration (see supplementary material for the full list).

### Age profiles of HC and LTC per capita cost

The age profiles of estimated HC and LTC cost in Norway are by and large in agreement with stylised facts for other european countries [6]. Based on our estimates, HC cost are higher than LTC cost until reaching the 80s. Similar results have been reported in e.g. Denmark and the Netherlands [7, 8]. Although Norway, like other Scandinavian countries, has highly developed LTC services within both home-based and institutional care [4, 9], we still find considerable differences to other Scandinavian countries. There is, for instance, a higher share of LTC in total costs in Norway compared to Denmark,^3^ the main cause being higher user rates of LTC in Norway [10]. This is, in particular, related to Norway having a high rate of non-elderly users [4, 10]. LTC costs are substantial in younger ages, amounting to 25–30% of total cost in most non-elderly age-groups.

Most HC and LTC services are skewed towards the elderly, reflecting an increased burden of illness and disability with increasing age [11, 12]. The difference in estimated total per capita cost between people in their 90s compared to people in their 60s is about 6.5 times for males and 9 times for females. This is comparable to the age differences found in a Dutch study [8]. A decreasing age curve in very high age observed for HC cost is also found in several other studies [7, 8, 13, 14], but not all [15, 16]. We can only speculate on why there are differning age-patterns for the oldest. One explanation could be differences in the medical care provided in residential care, but we do not have information to shed light on this.

There has in later years been much discussion on the interpretation and use of information about the age gratient in healthcare cost. Age may be a proxy for serveral underlying factors, including disability and proximity to death (ill health) which lead to higher costs [2]. Improvements in population health for older age groups may lead to naïve projections of future cost because cost estimates based on current cost by age-profiles will overstate the need for additional health spending due to population ageing. Studies on trends in aging populations have so far been unable to reach a consensus on wether there is an compression or expansion of morbidity in old age. This question seems to depend on, among other things, country, time period and the health indicator under study [11]. Disability-related or impairment-related measures of morbidity, e.g. ADL-functioning, tend to support compression of morbidity, whereas chronic disease morbidity tends to support the expansion of morbidity. This could indicate an increase in the future need of HC and decrease in need of LTC compared to naïve projections [17]. There is high prevalence of multimorbity among older adults, and this increases with age [18]. An increase in the prevalence and complexity of multimorbidity have been reported [19], which will drive costs up, especially in social and inpatient care [20]. Frailty and multimorbidity are two related conditions in older adults [21]. Frailty have received inreased attention as a geriatric syndrome, distinct from disability and comorbidity, as a way of understanding health diversity among older adults [22], and as a driver of healthcare costs in old age [23, 24]. It is potentially reversable, and amenable to prevention and remediation, and therefore a potentially important risk stratification indicator [25]. A recent Italian study found a non-linear effect of frailty level on hospital cost, and the highest level of hospital admisssions and cost among pre-frail older adults [26]. The risk of developing pre-frailty and frailty is high among older adults [27], and, thus, significant predictors of nursing home placement among community-dwelling older adults [28]. Pre-frailty is typically associated with initial psycho-physical impairment or lack of socio-economic resources, and therefore a potential target for effective and low cost interventions that could reduce costs substantially [26].

Mental healthcare accounts for over one fifth of total HC cost. This includes only specialist care, not related primary care or LTC cost at the municipal level. The high share of mental health in Norway seems to be among the exceptions rather than the rule [29]. A recent mapping of mental health services in eight study areas in Europe found a high level of resource use in the Norwegian study area [30]. Mental healh care represent an important exception to the general pattern of skewness of HC cost towards high age; per capita cost is higest among adolecents and young adults. Mental disorders account for a large proportion of the disease burden in young people [31], and especially in younger ages there are high prevalence of mental health illness among high-cost patients [32]. Poor mental health is strongly related to e.g. lower educational achievements, substance abuse, violence, and poor reproductive health [31], having detrimental effects on long-term life outcomes. Our study, is in this regard, yet another reminder of the importance of mental health services and preventive measures for the young generations.

### User rates and user costs

Althoug user rates are high for prescription medicine and primary care in all ages, user costs are low, and these services are therefore of limited importance in total cost. For LTC, on the other hand, user rates are low in younger ages (less than 5 % before retirement age), but user costs are high. LTC cost is therefore higher than the cost of both prescription medicine and primary care for non-elderly. The decrease in user costs for home help from the 30s is, in particular, related to the decrease in cost of training in activities of daily living. Furthermore, when moving into the older agebands the home care user population changes and more people with limited care needs enter. The typical younger, high-cost LTC user groups, e.g. neurological disorders like MS, intellectual disability, mental illness and substance abuse [33], also have shorter than average life expectancy [34–36]. Analyses of LTC cost are typically focussed on the elderly. Our results point to the importance of including LTC when adressing health related spending needs in the younger population as well. The relative number of younger LTC users has seen a sharp rise in Norway, while user rates for ≥67 year olds has been decling [37].

In the oldest age groups, HC and home-based LTC costs decrease due to drops in user rates, and, for HC services, a decrease in cost per user. Lower user rates of HC and home-based services in high age are mirrored by increased user rates for long-term nursing home care. Becoming a long-term resident in nursing homes in Norway implies that primary healthcare and medication are mainly covered by the nursing home. A study of nursing home residents in a large Norwegian municipality found that admission rates to hospital were lower for the oldest (90+) residents compared to community dwellers in the same age group [38]. Studies from other countries have found lower hospitalisation rates among nursing home patients as a group compared to their pre-nursing home period [39, 40], community counterparts [41], home care recipients [42], and those in special accommodation or assisted living facilities [43, 44]. A common assumption is that lower HC user cost among the very old is caused by lower treatment intencity [45–47], but our data suggest that this can, at least partly, be attributed to a higher share of users not having full year of HC costs. This occurs because these age groups have higher death rates, being in their final stage of life, or because they relocate into nursing homes during the year, and thereby don’t accrue the cost of full years. This is also augmented by the fact that people with high medical needs have higher risk of becoming nursing home patients [48–51].

### Gender differences

There are important gender-related differences in costs and our analysis has uncovered several of them. Over all, women use more health and care services than men and constitute more than 55% of total costs. Total per capita cost is higher for females than males in the ages between 15 and 55 and among the elderly 80+. Males have higher HC cost than females for ages 60+, while females had higher LTC cost than males after 70. Similar gender differences have been found in other countries [7, 8].

We were able to gain more neuanced insights into these gender differences by decomposing cost in user rates and user cost. Femals have higher user rates for HC than males in their reproductive ages, and almost twice the user rate of somatic health compared to males in their early 30s (i.e. when the fertility rate is highest). User costs of primary care and somatic HC are also higher for females at this age. Higher HC utilisation among females than males can be attributed not only to medical needs but also to social, cultural and behavioural factors such as help-seeking behaviour [52]. Higher user rates for females of HC services and also of the major home-based LTC services continues until the 70s or 80s. At higher ages females more often than males receive long-term residential care.

The health of males and and females differ [53]. The observed gender differneces in HC and LTC service use and cost can be seen in light of the ‘male-female health-survival paradox’; females live longer, but in poorer health, i.e. with higher burden of chronic disease and disability, than males [54]. This is reflected in the gender distribution of HC and LTC cost. After retirement age, an increasing share (> 50%) of HC and LTC spending within age-groups goes to females, reaching between 80 and 90% in centenarians. This mirror an increasing share of females in the old-age population (from 50% in 65–69 to > 80% in 100+).

Several biological (genetic, hormonal and immunological) and behavioral and social explanations (risk-related activities, illness perception, healthcare utilization, gender roles) for the gender-paradox have been suggested [55, 56]. One hypotesis is that females experience more chronic non-fatal and disabling conditions than males, leading to higher morbidity, while males experience more ‘life-threatening’ conditions resulting in higher mortality [55]. Excess mortality in males have historically been found in certain groups (ages 50–70) in large part due to gender differnces in cardiovasucular diseases and smoking behavior [57]. The gender-paradox fits well with higher HC utilisation rates in middle aged women, since high morbidity is related to high service use [58], but at lower cost per user in the same five-year age-bands than men. This corresponds to high mortality-related costs among middle aged men [2, 59].

Females seem to tolerate frailty better than males [55]. One explanation could be that greater physiological reserves may enable females to acquire more deficits in multiple organ systems without succumbing [60]. Females being more frail than males fits well with higher degree of LTC use and institutionalisation in older females compared to older males as seen in this study. Higher rates of nursing home placement among females are a common observation. A review of 11 studies found the admission rates to be between 40 to 60% higher for females than for males [61]. Again this could relate to both biological and social factors. Frailty is a significant predictors of nursing home placement [28]. Another important risk factor for insitutionalisation is dementia [62], which disproportionally affects females [63]. Gender differences in LTC could also relate to age differences between spouses, i.e. the risk of outliving the spouse [64], gender differences in the role as informal caregiver, and biological reasons for diffential responses to spousal bereavement [56].

A Norwegian study of home care recipients found that living alone was associated with more formal care. Male cohabitants, however, received less formal care than female cohabitans [65]. Widowhood is found to be a strong predictor of insitutionalisation [62]. A US study [66] found clear gender differences in nursing home placement in response to spousal death and also in the protective effect of having offspring. While the risk of nursing home placement for females were unaffected by spousal death, the risk doubled for males. In a similar vein, the effect of having children on nursing home placement were unrelated to the spousal status for women, but reduced the risk of nursing home placement for males only after the death of their wife. These results are in accordance with the finding in the previously mentioned review [61].

The gender gap in longevity is narrowing [56, 67, 68]. Several factors such as decreased mortality from ischemic heart disease (larger positive impact on male life expectancy), increased mortality from Alzheimer’s disease (larger negative impact on female life expectancy) and causes related to smoking (decreased in males, but increased in women) have contributed to shrink the gender gap in life expectancy [69].

There are also important gender differences in the utilsation and cost of mental healthcare. 51% of total cost of mental health care is spent on women. Females have higher user rates than males in most ages, except among the youngest (< 15) and the oldest (100+). User costs on the other hand were higher for males than females in ages 20–60, while the opposite is found for higher ages. A large survey of several European countries found higher overall mental healthcare use among females than men, although males with severe mental healthproblems (SMI) had significantly higher probability of service use compared to females with similar SMI and background characteristics [70]. Among females, both cost and user rates peek in the late teens (15–19 years). Among men, user rates peeks at 10–14 year and cost peaks in late twenties. A Danish study of incidence rates of treated mental disorders showed incidence rates peaking during adolecent or young adulthood [71]. The incidence rate around the peak was higher for males than females for schizophrenia and illnesses related to psychoactive substance abuse, while the reverse was found for mood disorders. The gender differnce was huge for e.g. eating disorders (female dominates) and for attention deficit hyperactivity disorder (ADHD) and autism spectrum disorder (ASD) (male dominates). The indicence rates of ADHD and ASD showed an early onset which is consistent with the finding in the present study that user rates for mental health care peaks in early teens for males. The study also showed that some dignoses e.g. schizophrenia, eating disorders, ADHD and ASD have incidence rates that declines rapidly with increaseing age, while other disorders e.g. psychoactive substance abuse and mood disorders do not. Declining user rates between ages 30 and 65 as we find in our study fits well with this incidence pattern. Also the age profile of treated insidence rate for mood disorders (bimodal distribution with an increase in old age (female dominance) and a decrease for the oldest) and of organic mental disorders like dementia (late onset, a sharp increase in old age with no clear gender differences and a decrease for the oldest) found in the Danish study are consistent with the leveling out of user rates after retirement age and a dip among the oldest in our study. 

### Strengths and limitations

The major strength of this study is that it covers most HC and LTC services for the entire Norwegian population, and provides not only the overall age and gender profile of total cost, but brings additional insights into the variation in service composition over the life-span, as well as decomposing per captia cost in user rates and user costs for a wide range of services. Norway is an particularly interesting case since it has a highly developed LTC service system both for home-based and institutional services together with high levels of mental health services.

A limitation in the study is that it was prevented from linking data between registers, and is therefore unable to estimate total user rates. Moreover, register data on care could not be linked to the population registry, which means that there may be slight inaccuracies in calulating the per capita measures. This could potentially affect results for small populations groups such as males in the 100+ age group totaling less than 200 persons.

The data were collected as part of a broader study involving a number of complex data requirements. Hence, the process of extracting the data from the different registries became protracted, partly because of the very strict privacy regulation of the LTC data and the lack of experience in the registries and regulating authorities in delivering this type of complex data to research. The data we use are therefore from the year 2010, which at the time of our first extraction application was relatively fresh.

## Conclusion

Demographic changes affect both the level and composition of health and care needs in the population. Information about the age and gender distribution of services and costs are vital for developing better and appropriate services both at the local and national level. The future development in the demand for HC and LTC and related costs depend on a complex set of biological, behavioural and social factors. The potential effect of policy is large. Compression of disability-related morbidity, i.e. postponement of functional limitations and disability, could dampen the effect of longer life-expectancy on the demand for LTC in the future. The expansion of chronic disease morbidity, on the other hand, could increase the demand for HC, but could also reduce death-related costs due to higher age at death. A shift towards more home-based LTC-services can increase the demand for HC as LTC in institutional settings reduces the use of hospital care and other HC servies. The narrowing gender-gap in longivety is another factor likely to drive such a development. Aging populations have drawn much attention to the rising health and care needs of elderly, but results here show that a significant part of the cost is still related to the younger population. Our study therefore underscores the need for an attentive and pro-active stance towards the high service prevalence and high cost of mental health care in our upcoming generations.

## Supplementary information

**Additional file 1.** Supplementary material on data sources, services included, units of measurement, cost estimation and unit costs, and service cost estimates compared with other sources.

## Data Availability

The analysis was based on register data. Data from the registries are available for research projects approved by the registries and that meets the requirements of the Health Research Act and the Personal Data Act, so the authors are not allowed to share the data. For data requests contact: service@helsedata.no.

## Abbreviations

ADHD: Attention deficit hyperactivity disorder
ADL: Activity of daily living
HC: Healthcare
ASD: Autism spectrum disorder
IPLOS: The Norwegian Information System for the Nursing and Care Sector
KUHR: The registry for Control and Payment of Health Refunds
LTC: Long-term care
MS: Multiple sclerosis
NorPD: The Norwegian Prescription Database
NPR: The Norwegian Patient Registry
RHA: Regional health authority
SHA: System of health accounts
US: United states

## Footnotes

1 In Table 1 we use four age-bands (0–19, 20–39, 40–64, 65–84, 85+).

2 Respite care, care salary, home help and activity.

3 In 2013 Norway spent 2.4% of gross domesitic product (GDP) on LTC and 8.9% of GDP on health, while Denmark spent 2.3% of GDP on LTC and 10.4% on health [10].
