# Supplementary material for: Health and care service utilisation and cost over the life-span: a descriptive analysis of population data
Source: BMC Health Serv Res. 2020 May 19;20:435. doi: 10.1186/s12913-020-05295-2 (PMC7236310; doi:10.1186/s12913-020-05295-2)
Supplement: Supplementary file 1 — Additional file 1. Supplementary material on data sources, services included, units of measurement, cost estimation and unit costs, and service cost estimates compared with other sources. [file 12913_2020_5295_MOESM1_ESM.docx]

**Supplementary material**

Most publicly funded services are included in the datasets used in this study. Exceptions include public dentists, physiotherapists, manual therapists and chiropractors, services for mother and children and school nurses, but these services do not account for a significant portion of the cost compared to the included services. Health and care services paid entirely by the users, such as most dental and optician services, non-prescription medicine and some private specialist services, are not included in available data bases. Also, therapeutically appliances and other medical durables, as well as ancillary services to healthcare, prevention and public health services and Health administration and health insurance are not included. Long-term care (LTC) comprises both health-related and social care services. Taking in to account the difference in LTC in SHA and other official statistics (see table S3 below), our cost estimate covers about 73% of the total healthcare cost in 2010, as measured in the Health Accounts of Statistics Norway.

Total population of Norway in 2010 was calculated as the population at January 1^st^ 2010 adding the number of births in 2010 and the number of immigrants in 2010.

**Table S1 Service utilisation. Data source, service and unit of measurement.**

| **Registry** | **Service** | **Utilisation – unit of measurement** |
| --- | --- | --- |
| **KUHR** a register for Control and Payment of Health Reimbursement | General practise, primary care emergency visits  Private specialists | Contact/Visit |
| **NorPD** Norwegian Prescription Database | Prescription drugs† | Dispensed prescriptions (AUP) |
| **NPR** Norwegian Patient Registry | Somatic hospital inpatient, daypatient, outpatient | DRG-points (cost calculation)  Days |
|  | Rehabilitation institutions  Psychiatric and substance abuse inpatient treatment | Days |
|  | Psychiatric and substance abuse outpatient treatment | Contacts |
| **IPLOS** the Norwegian Information System for the Nursing and Care Sector | Home nursing, practical assistance with activities of daily living, user-controlled personal assistance, care salary, respite care, support person, day center, day/night stay nursing home | Hours |
|  | Long term and short term residential care, meals on wheels, safety alarms | Days |

† Drug cost in hospitals and nursing homes are included in the cost estimates for the respective services.

**Table S2 Service cost estimation. Data source, service, unit of measurement, unit cost and source.**

| **Service** | **Data source^1^** | **Unit** | **Unit cost** | **Source unit cost** |
| --- | --- | --- | --- | --- |
| **Primary care physicians** | | | | |
| General practise | KUHR | Contact/ Visit | (Reimbursement and out of pocket) *1/0.75 | Assuming fee for service reimbursement and out of pocket cover 75% of costs (rest is coved by capitation), based on data from Deloitte (2011)^2^ |
| Emergency visits | KUHR | Contact/ Visit | (Reimbursement and out of pocket) *1/0.75 | Used the same formula as for ordinary practice |
| **Medicine** | | | | |
| Prescription drugs | NorPD | Dispensed prescriptions | Pharmacy retail price | From registry |
| **Specialised (secondary) healthcare** | | | | |
| Somatic inpatient | NPR | DRG-points | 44678 | Norwegian Directorate of Health (2011)^3^ |
| Somatic daypatient | NPR | DRG-points | 44678 | Same as somatic inpatient |
| Somatic outpatient | NPR | DRG-points | 44678 | Same as somatic inpatient |
| Somatic private specialist | KUHR | Contact/  Visit | (Reimbursement and out of pocket) *1/0.75 | Assuming fee for service reimbursement and out of pocket cover 75% of costs (rest is coved by operating subsidy), based on data from Deloitte (2011)^4^ |
| Rehabilitation institutions | NPR | Days | 2400 | Own calculation based on data from Norwegian Directorate of Health (2016) |
| Child and adolescence mental health inpatient | NPR | Days | 15079 | Based, with correction for price changes, on Norwegian Directorate of Health (2013) |
| Child and adolescence mental health daypatient | NPR |  |  | Excluded^5^ |
| Child and adolescence mental health outpatient | NPR | Contact/  Visit | 2821 | Own calculation based on data from Norwegian Directorate of Health |
| Adult mental health inpatient | NPR | Days | 8950 | Based, with correction for price changes, on Norwegian Directorate of Health (2013) |
| Adult mental health daypatient | NPR | Days | 4475 | Half of inpatient cost |
| Adult mental health outpatient | NPR | Contact/  Visit | 2238 | Based, with correction for price changes, on Norwegian Directorate of Health (2013) |
| Mental health private specialist | KUHR | Contact/  Visit | (Reimbursement and out of pocket) *1/0.50 | Assuming fee for service reimbursement and out of pocket cover 75% of costs (rest is coved by operating subsidy), based on data from Deloitte (2011)^6^ |
| Substance abuse inpatient | NPR | Days | 4378 | Based, with correction for price changes, on Norwegian Directorate of Health (2013) |
| Substance abuse daypatient | NPR | Days | 2189 | Half of inpatient cost |
| Substance abuse outpatient | NPR | Contact/  Visit | 2627 | Based, with correction for price changes, on Norwegian Directorate of Health (2013) |
| **Long term care^7^** | | | | |
| Home nursing | IPLOS | Hours | 580 | Based, with correction for price changes, on Vossius et al. (2015) |
| Home care - daily activity | IPLOS | Hours | 450 | Own calculation based on Norwegian Directorate of Health (2015) and Vossius et al. (2015) |
| Home care - training daily activities | IPLOS | Hours | 485 | Own calculation based on Norwegian Directorate of Health (2015) and Vossius et al. (2015) |
| Home care - user administered personal assistant | IPLOS | Hours | 295 | Based, with correction for price changes, on Norwegian Directorate of Health (2015) |
| Meals on wheels | IPLOS | Days | 35 | Based, with correction for price changes, on Vossius et al. (2015)+assumption that a user receives meals half of the days in the registered period, cf Oslo Economics 2017. |
| Safety alarm | IPLOS | Days | 7 | Based, with correction for price changes, on Vossius et al. (2015) |
| Care salary | IPLOS | Hours | 185 | Based, with correction for price changes, on Norwegian Directorate of Health (2015) |
| Respite care out of institution | IPLOS | Hours | 210 | Based, with correction for price changes, on Norwegian Directorate of Health (2015) |
| Activity - day centre etc | IPLOS | Hours | 135 | Based, with correction for price changes and assumption that average day price covers 6 hours per day, on Vossius et al. (2015) |
| Activity - Support person | IPLOS | Hours | 180 | Based, with correction for price changes, on Norwegian Directorate of Health (2015) |
| Long term residential care | IPLOS | Days | 2085 | Own calculation, average of several previous estimates from different sources |
| Short term residential care | IPLOS | Days | 2245 | Scaled estimate for long term residential care, based on calculated ratio of cost for long term and short term bed from Norwegian Directorate of Health (2015) |
| Respite care in institution < 20 years | IPLOS | Hours | 187.5 | From PWC/KS (2012) |
| Respite care in institution >= 20 years | IPLOS | Hours | 72.9 | From NOU 2011:17 |
| Day/night stay in institution | IPLOS | Hours | 86.9 | Based on estimate for cost per day for long term residential care |

1) KUHR a register for Control and Payment of Health Refunds, NPR Norwegian Patient Registry, NorPD Norwegian Prescription Database, IPLOS a register for Individual-Based (municipal) Health and Care Statistics.

2) State reimbursement and user charges account for about 75 percent of primary care physician costs on average and the rest is covers by municipal subsidy.

3) Calculated cost per DRG-point, excluding capital. This is not the same as the unit price used as basis for reimbursement from the state.

4) State reimbursement and user charges account for about 75 percent of private specialist costs on average and the rest is covers by state subsidy.

5) Problem with identifying the number of days spent in hospital in a period of admission as daypatient. Day activity small part of total activity.

6) State reimbursement and user charges account for about 50 percent of private psychiatric specialist costs on average and the rest is covers by state subsidy.

7) Service use is calculated based on information on start and end date of municipal decision to grant the service, combined with information on average hours per week for relevant services.

**Table S3 Service cost estimates compared with other sources. Costs 2010, million NOK**

| Service | This study | Health account, Statistics Norway | Comments,  Health account | Other sources | Comments,  Other sources |
| --- | --- | --- | --- | --- | --- |
| Somatic healthcare | 59.6 |  |  | 57.6 | Statistics Norway, Tabell: 06464 |
| Mental healthcare | 21.9 |  |  | 20.1 | Statistics Norway, Tabell: 06464 |
| Sum Spesialist healthcare | 81.5 | 83.0 | HC.1.1 + 1.2 + 1.3.3 + 2 | 77.7 |  |
| Primary care physician | 7.5 | 19.0 | HC.1.3.1  The number for 2010 is to high  since it includes also physiotherapists and chiropractors. It also includes physicians not included in the publicly paid system and other expenditures not covered in the KUHR data base. Our estimate is close to an alternative estimate based on average income for primary care physicians multiplies with the number of physicians. | 10.8 | Statistics Norway, Tabell: 04685, KOSTRA f241 Diagnose, behandling, re-/habilitering + KUHR. Primary care physicians are paid partly by the municipalities (included in KOSTRA f241) and by the state and patients (included in KUHR). Our estimate only cover activity in general practice and emergency visits. Excludes other primary physician activity in municipal healthcare (nursing homes, prison health, etc) included in KOSTRA f241 |
| Prescription drugs | 13.2 | 16.2 | HC.5.1.1 + 5.1.2 | 15.0 | = 13.2/0.88. Prescription drugs account for 88% of sales. Sakshaug, S (ed), Drug Consumption in Norway 2006-2010 (Legemiddelforbruket i Norge 2006-2010), Oslo, Norwegian Institute of Public Health, March 2011 |
| Long-term care | 70.1 | 65.8 | HC.3 | 70.3 | Statistics Norway, Tabell: 04686, Korrigerte brutto driftsutgifter KOSTRA f234+253+254+261 |
| Sum | 172.3 | 184.0 |  | 173.8 |  |
| Total HC |  | 230.8 |  |  |  |
| Not covered |  | 46.7 | HC.1.3.2 + 1.3.9 + 1.4 + 4 + 5.1.3 + 5.2 + 6+7 |  |  |

**References**

Deloitte (2011). Inntekts- og kostnadsundersøkelse (IKU) for driftsåret 2009 [Income and cost investigation for year 2009]. Oslo

Norwegian Directorate of Health (2011). Samdata Spesialisthelsetjenesten 2010 [Samdata Specialist healthcare], Report IS-1921.Oslo

Norwegian Directorate of Health (2016). Private aktører i spesialisthelsetjenesten. Omfang og utvikling 2010-2014 [Private providers in specialist healthcare. Volume and development 2010-2014]. Report IS-2450. Oslo

Norwegian Directorate of Health (2013). Utredning av modeller for kommunal medfinansiering av psykisk helsevern og rusbehandling (TSB) [Examination of models for municipal co-financing of mental healthcare and drug treatment]. Case-number 11/7866-14.

Norwegian Directorate of Health (2015). Forsøksordning med statlig finansiering av omsorgstjenester [Trial with state funding for long term care services]. IS-2392. Oslo

Oslo Economics (2017) Potensial for samfunnsøkonomiske gevinster innen matlevering til offentlig sector.

Vossius C. et al. (2015). Ressursbruk og sykdomsforløp ved demens (REDIC) [Resource use and disease progression in dementia]. Alderspsykiatrisk forskningssenter, Sykehuset Innlandet

PWC/KS (2012). Kommunale merkostnader som følge av Kaasa-utvalgets anbefalinger [Additional costs for the municipalites by implementing the recommendations of the Kaasa-expert committee ].

NOU 2011:17 "Når sant skal sies om pårørendeomsorg" [When telling the thruth about the care for relatives]. Report from the Kaasa-expert committee. 2011, Ministry of Health and Care Services.
